# Genomic epidemiology of *Streptococcus dysgalactiae* subsp. *equisimilis* strains causing invasive disease in Norway during 2018

**DOI:** 10.3389/fmicb.2023.1171913

**Published:** 2023-04-17

**Authors:** Alba Kaci, Christine M. Jonassen, Steinar Skrede, Audun Sivertsen, The Norwegian Study Group on Streptococcus dysgalactiae, Aasmund Fostervold, Martin Steinbakk, Oddvar Oppegaard

**Affiliations:** ^1^Center for Laboratory Medicine, Østfold Hospital Trust, Grålum, Norway; ^2^Department of Virology, Norwegian Institute of Public Health, Oslo, Norway; ^3^Department of Clinical Science, University of Bergen, Bergen, Norway; ^4^Department of Medicine, Haukeland University Hospital, Bergen, Norway; ^5^Department of Microbiology, Haukeland University Hospital, Bergen, Norway

**Keywords:** whole genome sequencing (WGS), SDSE, surveillance studies, genomics, horizontal genetic transfer

## Abstract

**Background:**

*Streptococcus dysgalactiae* subspecies *equisimilis* (SDSE) is an emerging global pathogen, yet the epidemiology and population genetics of SDSE species have not been extensively characterized.

**Methods:**

We carried out whole genome sequencing to characterize 274 SDSE isolates causing bloodstream infections obtained through national surveillance program in 2018. We conducted multilocus sequence typing (MLST), *emm*-typing, core genome phylogeny, as well as investigated key features associated with virulence. Moreover, comparison to SDSE from other geographic regions were performed in order to gain more insight in the evolutionary dynamics in SDSE.

**Results:**

The phylogenetic analysis indicated a substantial diversity of *emm*-types and sequence types (STs). Briefly, 17 *emm*-types and 58 STs were identified that formed 10 clonal complexes (CCs). The predominant ST-types were ST20 (20%), ST17 (17%), and ST29 (11%). While CC17 and CC29 clades showed a substantial heterogeneity with well-separated *emm*-associated subclades, the CC20 clade harboring the *stG62647 emm*-type was more homogenous and the most prevalent in the present study. Moreover, we observed notable differences in the distribution of clades within Norway, as well as several disseminated CCs and also distinct geographic variations when compared to data from other countries. We also revealed extensive intra-species recombination events involving surface exposed virulence factors, including the *emm* gene important for phylogenetic profiling.

**Conclusion:**

Recombination events involving the *emm* as well as other virulence genes in SDSE, are important mechanisms in shaping the genetic variability in the SDSE population, potentially offering selective advantages to certain lineages. The enhanced phylogenetic resolution offered by whole genome sequencing is necessary to identify and delimitate outbreaks, monitor and properly characterize emerging strains, as well as elucidate bacterial population dynamics.

## Introduction

*Streptococcus dysgalactiae* subspecies *equisimilis* (SDSE) is a β-hemolytic streptococcal species harboring the Lancefield groups C or G antigen (rarely group A or L) (Brandt et al., 1999; Rantala, 2014). The clinical manifestations of SDSE in humans resemble those caused by *Streptococcus pyogenes* (Group A Streptococcus; GAS), ranging from tonsillitis or harmless superficial skin infections to life-threating necrotizing soft tissue infections or streptococcal toxic shock syndrome (Brandt and Spellerberg, 2009; Broyles et al., 2009; Rantala, 2014; Bruun et al., 2016; Oppegaard et al., 2016). Although SDSE was long considered a low-virulent or commensal organism, increasing rates of invasive infections have been reported worldwide the past decades, exceeding the incidence rates of invasive *S. pyogenes* disease in some geographic regions (Rantala et al., 2009; Wong et al., 2009; Harris et al., 2011; Lambertsen et al., 2014; Schwartz et al., 2014; Wajima et al., 2016). In western Norway, the annual incidence rates for SDSE bloodstream infections gradually rose from 1.4 to 7.6 per 100,000 inhabitants during 1999–2021, and it is currently the fifth most common pathogen identified in blood cultures in this region (Oppegaard et al., 2015, 2023).

The cell surface M protein, encoded by the *emm*-gene, is a major virulence factor in both SDSE and *S. pyogenes*, and important in inhibiting phagocytosis and inactivation of the complement system (Bisno et al., 1987; Brandt and Spellerberg, 2009). It is ubiquitous in SDSE of human origin, and its hypervariability in the 5’-terminal region forms the basis of classification into *emm*-types. Moreover, SDSE is often classified using multilocus sequence typing (MLST), and to date, more than 100 SDSE *emm*-types and 600 sequence types (ST) have been described (CDC Strep Lab, PubMLST).

Despite SDSE being an emerging human pathogen, the epidemiology and population genetics of SDSE species are still poorly understood. The majority of published population studies have involved molecular characterization and typing by PCR methods, providing a limited phylogenetic resolution. The substantial diversity of circulating *emm*-types and STs observed in such studies, combined with an incongruence between these two phylogenetic markers, have complicated the interpretation of evolutionary relationships of SDSE isolates (Ahmad et al., 2009; McMillan et al., 2011; McNeilly and Mcmillan, 2014).

Whole genome sequencing (WGS) of bacterial pathogens has emerged as a powerful tool, providing higher resolution than those of conventional methods (Reuter et al., 2013; Lytsy et al., 2017). Systematic large-scale population-based surveillance of SDSE isolates using whole genome sequencing may therefore contribute to enhance our understanding of molecular epidemiology and pathogenic mechanisms of SDSE.

In the present study, we employed WGS to explore the epidemiological, evolutionary and molecular characteristics of clinical SDSE isolates obtained from a nationwide surveillance in Norway in 2018. In addition, we compared our data to other SDSE isolate-sequences retrieved from other geographic regions in the recent years to gain a global perspective and increase our understanding of the epidemiology of SDSE.

## Materials and methods

### Surveillance and bacterial isolates

All SDSE isolates from blood cultures in Norway during 2018 were collected as part of the Norwegian surveillance program for antimicrobial resistance (NORM). Collection of data within NORM follows a standard protocol described in the yearly surveillance reports (NORM/NORM-VET, 2018). Primary laboratories performed species identification based on colony morphology (β-hemolytic reaction on 5% sheep-blood agar and colony size >0.5 mm after 24 h of incubation), serogroup specificity using rapid Lancefield agglutination test, and matrix-assisted laser desorption/ionization time-of-flight mass spectrometry (MALDI-TOF MS). All isolates identified as *S. dysgalactiae* were submitted to Haukeland University Hospital, Bergen, or Østfold Hospital Trust, Grålum for susceptibility testing and genomic characterization. The patients’ residential postal code, as well as specimen type and date of collection were reported for each isolate. Annual incidence rates for SDSE bloodstream infections were calculated using population data from 2018 obtained from Statistics Norway.^1^

### Whole genome sequencing

At Haukeland University Hospital, genomic DNA was extracted from 119 isolates (collected from regions North, Middle and West), followed by sequencing on an Illumina 4000 HiSeq system (Illumina, San Diego, CA) to produce 150 bp paired-end reads as previously described (Oppegaard et al., 2017b).

The Ion Torrent technology and Ion S5XL system (Thermo Fisher Scientific, Waltham, MA, USA) was used by Østfold Hospital Trust to WGS the remaining 157 isolates collected in the South and East regions. Briefly, genomic DNAs were extracted from an overnight culture pre-treated with lysozyme and proteinase K using the QIAmp DNA Mini Blood extraction kit (QIAGEN, Hilden, Germany). Genomic libraries (300-base reads) were prepared using the Ion Xpress Plus Fragment Kit (Thermo Fisher Scientific, Waltham, MA, USA) according to the manufacturers’ instructions. Prior to template preparation and sequencing, all libraries were amplified, quantified by qPCR and pooled together to a concentration of 50 pM. Amplified and pooled samples were then sequenced on the Ion S5XL platform as described in the Ion S5 and Ion S5XL instrument user guide, using Ion 530 chips (Thermo Fisher Scientific, Waltham, MA, USA), with 32 samples per chip.

### *In silico* analysis

For data generated on the Illumina HiSeq system, reads were trimmed with Trimmomatic v0.39 (Bolger et al., 2014). For Ion Torrent-generated data, reads were processed with the incorporated S5 software plug-ins. All trimmed reads from the sequenced isolates were *de novo* assembled by SPAdes v5.14 (Bankevich et al., 2012), followed by evaluation of the assembled contigs using Quast v5.2.0 (Gurevich et al., 2013) and genome annotation using RAST v1.073 (Aziz et al., 2008). Species identity was confirmed by 16S rRNA analysis. Next, *emm*-types were determined by nucleotide BLAST-searches against the *emm* sequence database from the Center of Disease Control.^2^ MLST profiles were identified using the MLST-typing web-tool provided by the Center for Genomic Epidemiology (Larsen et al., 2012), and novel MLST alleles and profiles were submitted to pubMLST.^3^ STs were delineated into clonal complexes (CCs) based on single locus variants using Phyloviz (Ribeiro-Gonçalves et al., 2016). A core genome single-nucleotide polymorphism (SNPs) phylogeny was generated by Conserved Signature Indels (CSI) Phylogeny at the Center for Genomic Epidemiology^4^ using default settings and the SDSE type strain NCTC13762 as a reference.^5^ The resulting maximum likelihood phylogenetic trees were visualized using the Interactive Tree of Life platform, iTOL v6 (Letunic and Bork, 2021).

Geneious Prime v2022.2.2 was used to screen for the presence of key virulence factors and regulators, adapted and modified from the dataset of virulence factors of *S. pyogenes* in the Virulence Factors of Pathogenic Bacteria Database.^6^ *In silico* screening was performed with BLAST search using an E-value of 0.05 as cut-off. For identification of the hypervariable pilus islands we used BLAST to locate the conserved genes flanking these operons, and subsequently manually inspected interposing region for the presence or absence of *pilus operon* genes, including regulators, ancillary proteins, pilus tip adhesins and sortase genes. The genome of SDSE strains was further screened for bacteriophage sequences using PHASTER (Arndt et al., 2016) and Integrative Conjugative Elements (ICEs) were identified by manual inspection and BLAST-search of selected regions as previously described (Ambroset et al., 2015).

Whole genome sequences from epidemiological studies in Japan and Canada were downloaded from GenBank (PRJDB12179 and PRJNA325743). In order to match the disease severity and temporal characteristics of our study, only invasive isolates collected between 2012 and 2019 were included from GenBank. These genomes were *de novo* assembled (SPAdes), annotated (RAST) and MLST-typed (Center for Genomic Epidemiology). The geographical distribution of SDSE clonal complexes was visualized using Phyloviz.

## Results

### Patient characteristics and genome statistics of SDSE isolates

A total of 276 blood culture isolates were collected during the surveillance period in 2018 in Norway; 274 of the 276 blood culture isolates were confirmed as SDSE by whole genome sequencing and included for further analysis, whereas two isolates were identified as *S. canis* and *S. equi* and excluded from the study. The Lancefield group C antigen was detected in 81 isolates (30%) and 193 isolates were classified as Lancefield group G (70%). No isolates harbored group A or L-antigens. In general, a male predominance was observed (66%), and the age distribution ranged from 12 to 98 years; with a median age of 76 years.

The draft genomes of the 274 SDSE isolates had an average assembly length of 2.16 Mb; a GC content of approximately 39.2%; contigs >500 bp of 70; and protein encoding genes (CDSs) of 2,192 (genome annotation data and accession numbers are shown in Supplementary Table 1).

### Molecular typing and phylogeny

Genotyping revealed substantial diversity, comprising 17 *emm*-types in total (Supplementary Table 1). The most prevalent *emm*-types were *stG62647* (19%), *stG485* (13%), *stC74a* (12%), *stG6* (9.5%), *stG643* (8.3%), and *stG65*2 (5.8%). Among them, *emm*-types *stG6*, *stG643*, and *stG652* were composed of two or more subtypes. Seven new *emm*-subtypes were identified (*stC9431.1*, *stC485.19*, *stG6.23*, *stG6.24*, *stG6.25*, *stG2078.1*3, and *stG652.21*). The *emm*-type *stG62647* was exclusively detected in group C isolates, whereas *stC74a* was found restricted to group G isolates. The other prevalent *emm*-types included a mix of isolates expressing group G or C antigens.

**FIGURE 1:**
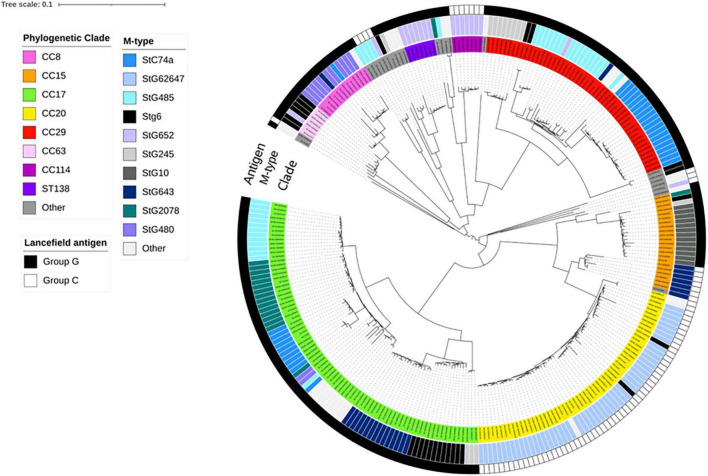
Whole genome single-nucleotide polymorphism (SNP)-derived phylogenetic tree of blood culture *Streptococcus dysgalactiae* subspecies *equisimilis* (SDSE) isolates obtained in Norway during 2018. This tree was constructed with CSI phylogeny website and metadata was added using iTOL, including information about antigen-type, *emm*-type and multilocus sequence typing clonal complex relatedness groups (CCs). Scale bar indicates the estimated evolutionary divergence between isolates measured in substitution per site. *N* = 273; excluding the canine-associated genome harboring the *stC9431 emm* gene. The first ring (Antigen) indicates the distribution of Lancefield group C or G antigen among the isolates. The second ring (M-type) indicates the distribution of SDSE isolates into *emm*-types, whereas the third ring (CC) indicates the most common clonal complexes and sequence types (STs).

Exploring the phylogenetic relationships, the one strain belonging to the *stC9431 emm*-type was separated from all other isolates by >14,000 SNPs (Supplementary Figure 1). Comparison to previously characterized SDSE-strains of animal origin revealed that it clustered with canine-associated strains (Porcellato et al., 2021). This outlier affected the ability to discriminate the remaining isolates, and a decision was made to perform phylogenetic analyses without this genome. A phylogenetic tree excluding the canine-associated strain is depicted in Figure 1.

**FIGURE 2:**
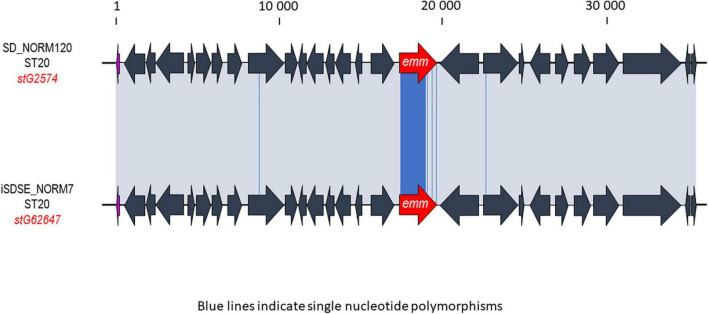
Graphic representation of a likely *emm*-switch event observed between two phylogenetically closely related isolates. The ST20 profile is usually associated with the *stG62647 emm* gene, but here the SD_NORM120 isolate harbors the *stG2574 emm* gene. The iSDSE_NORM7 strain was the closest relative in the single-nucleotide polymorphism (SNP)-analysis, and was chosen for comparison. The alignment was performed using Geneious. The *emm* gene in each *Streptococcus dysgalactiae* subspecies *equisimilis* (SDSE) isolate is highlighted in red. The gray shaded areas indicate 100% homology, and blue lines in the alignment indicate single nucleotide polymorphisms. The scale indicates number of base pairs.

Multilocus sequence typing performed for all SDSE isolates revealed a total of 58 STs, of which 16 new alleles and nine novel profiles. The predominant ST-types were ST20 (20%), ST17 (17%), and ST29 (11%). The ST-types clustered into ten clonal complexes and 13 singletons using single locus variants. The phylogenetic analysis indicated a substantial heterogeneity within the CC17 and CC29 clades, with CC17 isolates differing by a median of 1,337 SNPs (range 0–2,669), and CC29 isolates by a median of 1,070 SNPs (range 12–2,519). CC17 and CC29 were associated with numerous *emm*-types, but these were distributed into well-separated *emm*-associated subclades within the clonal complexes (Figure 1). The CC20 clade was more homogenous, and the isolates were separated by a median of 143 SNPs (range 0–1,006). Only five isolates were associated with other *emm*-types than *stG62647*. Inspecting the M- protein region in these five isolates we detected a recombinational replacement of the *emm* gene, whereas the surrounding region was almost identical to their phylogenetic background (Figure 2). We observed such *emm* switching events in CC20 isolates involving the acquisition of *stG2574* (two isolates), *stG6* (two isolates), and *stG5420* (one isolate) *emm* genes. On the other hand, *emm* gene replacements in CC17 and CC29 clades were associated with more extensive phylogenetic discrepancies, reflecting more established subclades (data not shown).

In order to capture the heterogeneity in the CC17 and CC29 clades, we decided to further delineate these CCs into *emm*-specific subclades in the following phylogenetic analyses.

### Phylogeography

The annual incidence rates for bloodstream infections varied geographically in Norway during 2018. Health Region North recorded the highest incidence rate of 7.6 cases per 100,000 inhabitants/year, followed by Health Region South with 6.2/100,000, Health Region Middle with 5.0/100,000, Health Region East with 4.9/100,000, and Health Region West with 4.2/100,000.

**FIGURE 3:**
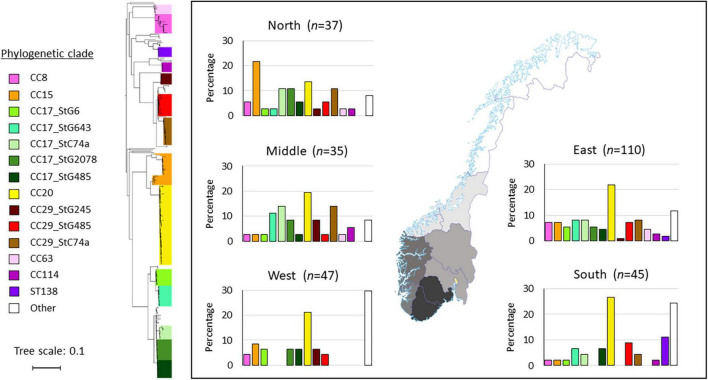
Distribution of *Streptococcus dysgalactiae* subspecies *equisimilis* (SDSE) clades and subclades in the five health regions in Norway. The geographic delineation of the regions is depicted on the map. In the histograms, the various clades and subclades are presented on the x-axis, and the y-axis represents the percentage of isolates within each region. The phylogenetic tree is constructed from single-nucleotide polymorphism (SNP)-analysis using the CSI-phylogeny website, and the clades and subclades were named based on multilocus sequence typing (MLST) and *emm*-type data. The tree scale bar indicates the estimated evolutionary divergence between isolates measured in substitutions per site.

Interestingly, geographic variations in the distribution of clonal complexes were also noted (Figure 3). The CC20 clade was dominant in all geographic regions, apart from the northern health region where CC15 was most common. Notably, the ST138 clade seems to have emerged in the south, constituting the second most frequent ST-profile in this region, whereas it was completely absent from the western, middle and northern regions. The western health region lacked several of the most common clades and subclades circulating in Norway. Approximately a third of the isolates in this region (14/47) belonged to less common clades, comprising nine different ST-profiles.

### Virulence profiling

To gain insight into genetic features promoting virulence, we investigated the presence of major SDSE-protein virulence factors (Table 1). All isolates harbored genes for the toxins Streptolysin O (*slo*), Streptolysin S (*sagA-H*), and Streptokinase (*Skc*), the adhesins *lmb*, *fnbB*, *fbp54* and *gapC*, as well as the immune modulators C5a peptidase (*scpA*) and Protein G (*prg*). The superantigens *ssa*, *smeZ*, *speA*, *speC*, *speH, speI*, *speJ*, *SpeK*, *speL*, *speM*, *speQ*, and *speR* were universally absent among all isolates, and *speG* was the only streptococcal superantigen detected (present in 61% of the isolates). The canine-associated genome (*stC9431*) did not harbor *slo*, *Skc*, *lmb*, or *scpA*. However, it was equipped with the immunoglobulin cleaving enzyme EndoS2D (*ndoS2D*), which was absent from all the other SDSE isolates.

**TABLE 1:** Distribution of the virulence genes in 274 invasive *Streptococcus dysgalactiae* subspecies *equisimilis* (SDSE) isolates collected in Norway during 2018.

Function	Gene	Description	GenBank ID	Present *n* (%)	MGE
Adhesion	*fnbA*	Fibronectin-bind	Z22150	0	–
	*fnbB*	Fibronectin-bind	Z29088	272 (99)	–
	*gfba*	Fibronectin-bind	U31115	123 (45)	–
	*fbp54*	Fibronectin-bind	L288918	274 (100)	–
	*gapC*	Plasminogen-bind	X97788	274 (100)	–
	*PI_1*	Pilus island 1/FCT	–	274 (100)	–
	*PI_2*	Pilus island 2	–	257 (94)	–
	*lmb*	Cell-adhesion	AB040535	273 (99)	–
	alp*	Cell-adhesion	VTT08026	49 (18)	Yes
	*bspA*	Cell-adhesion	CAD46802	121 (44)	Yes
Dissemination	*Skc*	Streptokinase	K02986	273 (99)	–
	*hylD*	Hyaluronidase	BAH81156	274 (100)	–
	*sda1*	DNAse	WP032463908	0	Yes
	*sda2*	DNAse	WP002988811	31 (11)	Yes
	*sdn*	DNAse	AAM80016	5 (2)	Yes
	*spd1*	DNAse	NP_268944	0	Yes
	*spd3*	DNAse	NP_269520	10 (4)	Yes
	*spd4*	DNAse	AAM79702	0	Yes
Immune evasion	*scpA*	C5a-peptidase	J05229	273 (99)	–
	*drsG*	AMP-cleave	AB508817	70 (26)	–
	*endoSD*	Ig-cleave	KT030921	0	–
	*endoS2D*	Ig-cleave	KT071707	1 (1)	–
	*prg*	Ig-binding/Protein G	Y00428	273 (99)	–
	*hasA*	Capsule	L20853	0	–
	*hasB*	Capsule	WP009880737	0	–
	*hasC*	Capsule	WP010922799	274 (100)	–
Toxin	*sagA-H*	Streptolysin S	AY033399	274 (100)	–
	*slo*	Streptolysin O	BAH82508	273 (99)	–
	*speA*	Superantigen	WP009880239	0	Yes
	*speC*	Superantigen	AAK33664	0	Yes
	*speG*	Superantigen	AF124499	168 (61)	–
	*speH*	Superantigen	AF124500	0	Yes
	*speI*	Superantigen	AF438524	0	Yes
	*speJ*	Superantigen	WP011284554	0	–
	*speK*	Superantigen	WP011054728	0	Yes
	*speL*	Superantigen	WP011017837	0	Yes
	*speM*	Superantigen	WP011017838	0	Yes
	*speQ*	Superantigen	BK010655	0	–
	*speR*	Superantigen	BK010662	0	–
	*ssa*	Superantigen	L29565	0	Yes
	*smeZ*	Superantigen	NP_269959	0	–
Other	*sil*	Invasive locus	KF188416	177 (65)	–

*Included the genes *alp3* and *dysalp*. MGE, mobile genetic element; FCT, Fibronectin Collagen and T-antigen.

**FIGURE 4:**
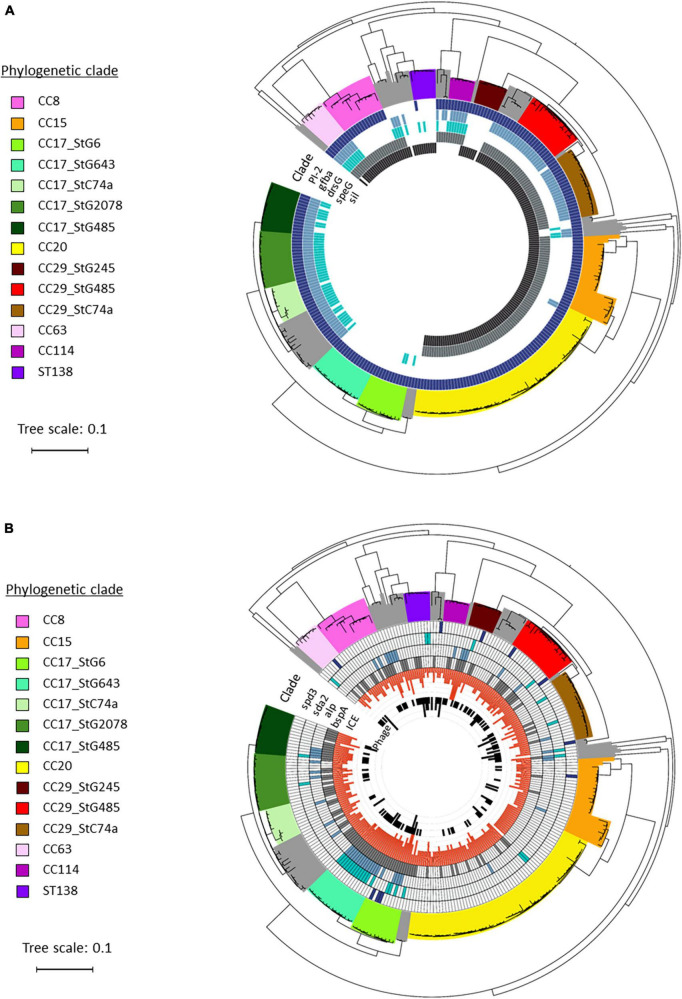
Distribution of virulence genes in blood culture *Streptococcus dysgalactiae* subspecies *equisimilis* (SDSE) isolates identified in Norway during 2018. Phylogenetic tree depicting the disposition of virulence genes among the SDSE isolates investigated in this study, and grouped in phylogenetic clades and subclades based on multilocus sequence typing (MLST) and *emm*-type data. The tree scale bar indicates the estimated evolutionary divergence between isolates measured in substitutions per site. **(A)** Distribution of chromosomal virulence factors, including Pilus island 2 *PI_2*, *gfba*, *drsG*, *speG*, and *sil*. **(B)** Distribution of virulence factors associated with mobile genetic elements, including phages and Integrative Conjugative Elements (ICEs). The phages associated DNAses *spd3* and *sda2*, and ICEs associated adhesins *alp* and *bspA* were detected among the different SDSE isolates.

**FIGURE 5:**
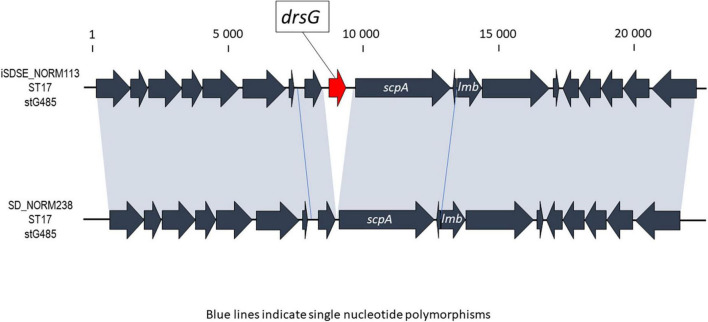
Graphic presentation of the event involving excision or insertion of the *drsG* gene in the C5a peptidase region of two phylogenetically closely related *Streptococcus dysgalactiae* subspecies *equisimilis* (SDSE) isolates. In the alignment, the *drsG* gene is marked in red, the gray shaded areas indicate 100% homology, and blue lines represent single nucleotide polymorphisms. The scale indicates number of base pairs.

A few chromosomally located virulence factors had a variable presence, including *drsG* (28%), *gfba* (45%), Pilus island 2 (*PI_2*) (94%), and *Streptococcus* invasive locus (*sil*) (65%). Distribution of these genes was highly linked to phylogenetic origin. However, this was occasionally reflected at the subclade level, and not necessarily congruent with ST or *emm*-profiles (Figure 4A). Interestingly, the virulence gene *drsG* had a more variable presence even within phylogenetic subclades, in particular in isolates from CC17. Inspecting the C5a-peptidase regions in these closely related CC17 isolates, we detected an excision or insertion of the *drsG* gene in some genomes (Figure 5). The surrounding genetic context was virtually identical.

Observed replacement of the Fibronectin, Collagen and T-antigen (FCT)-region (also known as the Pilus island-1 region) in addition to the *emm* gene (Supplementary Figure 2). These isolates had acquired a completely novel FCT-region harboring the *gfba* adhesin but were virtually identical to their phylogenetic ancestors in the surrounding genetic regions.

SDSE subspecies *equisimilis* isolates were also screened for mobile genetic elements (MGEs) and associated virulence genes (Table 1). The number of intact bacteriophages was low, on average 0.6 bacteriophages per genome (range 0–3). In line with this, we did not detect any phage-related superantigens, but some isolates harbored phages-associated DNAses, including *sda2* (11%), *spd3* (4%), and *sdn* (2%). Conversely, the prevalence of Integrative Conjugative Elements (ICEs) was relatively high among the SDSE isolates, with an average of 2.9 ICEs per genome (range 0–6). The ICE-associated virulence factors *bspA* and the *alp*-gene family, important for cell adhesion, were detected in 44% and 18% of the SDSE genomes, respectively.

Overall, these virulence genes carried by MGEs appeared to be randomly distributed, although an association with a few phylogenetic subclades was observed (Figure 4B).

### Geographical impact of SDSE clonal complexes

**FIGURE 6:**
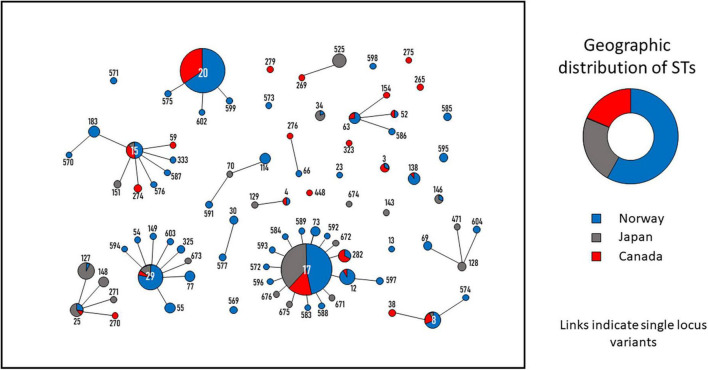
Minimum spanning tree depicting the genetic relatedness of *Streptococcus dysgalactiae* subspecies *equisimilis* (SDSE) blood culture isolates from Norway, Canada and Japan. The tree was constructed in Phyloviz. ST-profile and connections indicate single locus variants. The circles and numbers represent the individual sequence types (ST)-profiles. The size of the circles is proportional to the quantity of isolates belonging to each ST. The pie charts in the ST-circles indicate the percentage of isolates originating from the three countries. The donut chart shows the geographic distribution of all SDSE isolates included in the analysis. Blue = Norway; Red = Canada; Gray = Japan.

Next, we examined the geographic distribution of invasive SDSE clades by comparing our ST profiles to whole genome sequenced isolate collections from Canada (comprising 89 invasive isolates from 2012 to 2014) (Lother et al., 2017) and Japan (111 invasive isolates from 2012 to 2019) (Shinohara et al., 2023). Among the SDSE isolates retrieved from Norway, Canada and Japan, 34 CCs were identified. The CC8, CC15, CC17, CC25, and CC29 clades were among the most prevalent in all three continents, indicating that several of the major genotypes of SDSE have an intercontinental distribution (Figure 6).

Interestingly, CC20, which had the highest prevalence in Norway and Canada, was not present in the invasive isolate collection from Japan. Conversely, CC25 is frequent in Japan, whereas very few isolates of this CC were recovered in Norway and Canada. In general, the distribution of ST-profiles circulating in Norway and Canada was quite similar, whereas Japan seemed to have a more diverging phylogenetic SDSE population (Figure 6 and Supplementary Table 2).

## Discussion

*Streptococcus dysgalactiae* subspecies *equisimilis* (SDSE) is increasingly recognized as an emerging human pathogen implicated in invasive infections worldwide, yet the evolutionary mechanisms behind this development are still largely unknown. In this study, we characterized the genomic features of 274 invasive SDSE isolates recovered from a nationwide-based surveillance system in Norway during 2018. To the best of our knowledge, this is the first large in-depth genomic characterization by WGS of invasive SDSE isolates from a confined temporal and geographic population-based setting.

We observed a substantial diversity of *emm*-types and STs, as well as an imperfect association between *emm*-type and CC, indicating a high level of genomic plasticity in this species. Horizontal genetic transfer of the *emm* gene has been postulated to be the major driving force behind this population diversity in SDSE, but whole genome sequence data to support this has been lacking (Ahmad et al., 2009; McMillan et al., 2010, 2011). In the present study, we document several likely recombination events involving the *emm* gene in phylogenetically closely related SDSE isolates (Figure 2). Similar *emm* gene replacements have been noted in *S. pyogenes* in recent large-scale WGS studies (Chochua et al., 2017; Buckley et al., 2020), but the specific mechanisms involved in these horizontal genetic transfer events have not been elucidated. We did not detect any transposons or other mobile genetic elements in the vicinity, and transformation is the most likely mode of acquisition. However, an ability for natural transformation in SDSE and *S. pyogenes* remains to be shown (Mashburn-Warren et al., 2012).

Moreover, replacement events involving the FCT regions were also recorded between isolates. Recombination events in this locus have been reported, but predominantly in the form of intragenic mosaicism (McNeilly and Mcmillan, 2014). The FCT-region encodes the pilus apparatus (including the T-antigen) in *S. pyogenes* and SDSE, and mediates adhesion to host cells (Mora et al., 2005). Both the T-antigen and the M protein elicit strong antibody responses in humans, and replacement of these domains likely confers a selection advantage for the pathogen through immune escape (Mora et al., 2005). Recombination and exchange of these major immunogenic determinants thus appears to be important in shaping the evolution of the *S. pyogenes* and SDSE genomes.

In line with this, continuous replacement of circulating *emm*-types has been noted in *S. pyogenes*, and several outbreaks with novel virulent *S. pyogenes* clades have been characterized (Colman et al., 1993; Sanson et al., 2015; Zhu et al., 2015). The population dynamics of SDSE have been studied to a lesser extent, but the emergence of the CC20 clade harboring the *stG62647 emm*-type was reported in Norway few years ago (Oppegaard et al., 2017a). This clade has continued to expand, and it was the most prevalent genotype observed in the present study. The *stG62647 emm*-type appears to have gained a strong foothold also in other European countries, and recent studies in Austria (Leitner et al., 2015), Spain (Rojo-Bezares et al., 2021), Germany (Rößler et al., 2018), and Denmark (Lambertsen et al., 2014) all reported *stG62647* as the predominant circulating *emm*-type. Moreover, the CC20_*stG62647* clade comprised 31% of the SDSE isolates in Canada during 2012–2014, suggesting an intercontinental spread (Lother et al., 2017). In contrast, both CC20 and *stG62647* are infrequent in Japan, and the CC17_*stG6792* has been reported as the major clade in this region since 2003 (Wajima et al., 2016; Ikebe et al., 2018).

The CC20 was characterized by a high genetic homogeneity in our study, likely indicating a relatively novel clade. It was almost exclusively associated with the *stG62647 emm*-type, and only two isolates had single allele mutations giving rise to novel STs within the clonal complex. Differently, the CC17 and CC29 comprised several STs, and were associated with numerous *emm*-types, suggesting more established CCs (Figure 1). Nevertheless, the CC17 and CC29 isolates were distributed in well-separated *emm*-associated subclades within the clonal complexes, likely reflecting an *emm* switch event in a distant ancestor that has subsequently evolved into successful subclades over time. As such, neither *emm*-typing nor ST-profiling accurately captures the complexity of the phylogenetic relationships in SDSE. Insufficient phylogenetic resolution of these traditional typing tools has previously also been noted in *S. pyogenes*, highlighting the need for whole genome analyses to unravel the finer details of epidemiological trends in bacteriology (Davies et al., 2019).

Studying the distribution of clades within Norway, there were notable differences even in this confined geographic and temporal setting (Figure 3). Similarly, comparing the SDSE phylogeny in Norway to Japan and Canada, we observed several intercontinental disseminated CCs, but also distinct geographic variations (Figure 6). Taken together, our findings reflect the dynamic evolution of the SDSE population, where continuous rise and fall of novel lineages leads to dissemination of successful clones, but also large temporospatial differences in circulating SDSE strains.

Similar to previous reports, we found that the majority of virulence factors previously characterized in SDSE were ubiquitous, including genes involved in adhesion (*lmb*, *fnbB*, *fbp54*, and *gapC*), toxins (*slo*, *sagA-H*, and *Skc*) and immune invasion (*scpA* and *prg*) (Rantala, 2014; Wajima et al., 2016; Lother et al., 2017; Oppegaard et al., 2017b; Rößler et al., 2018; Rojo-Bezares et al., 2021). However, for the few chromosomally encoded virulence genes with variable presence, we observed clonally restricted virulence profiles in the SDSE isolates (Figure 4A), a finding that was also reported in a recent whole genome sequencing study from Canada (Lother et al., 2017).

Several studies have noted an association between the superantigen *speG* and certain *emm*-types, including *stG62647* and *stC74a* (Kittang et al., 2011; Leitner et al., 2015; Oppegaard et al., 2017b). We found *speG* to be present in isolates of the *stC74a emm*-type belonging to CC29, but not to CC17 (Figure 4A). Moreover, *speG* was absent in CC29 isolates harboring the *stG245 emm*-type, indicating a distribution in accordance with the phylogenetic subclades, but not necessarily in accordance with *emm*-type nor CC. This infers that recombination events involving acquisition or loss of *speG* gene have occurred in the evolution of these subclades.

In line with this, we observed several events of insertion or deletion of the *drsG* gene in closely related CC17 isolates (Figure 5), highlighting that recombination events also are apparent in SDSE in virulence genes other than the *emm* gene. DrsG is a homolog of streptococcal inhibitor of complement (SIC) and distantly related SIC (DRS) proteins secreted by *S. pyogenes*, playing an important role in inhibiting complement-mediated lysis and/or the activity of certain antimicrobial peptides (McNeilly and Mcmillan, 2014). In SDSE, this gene resides next to the *scpA* gene, a region that has been demonstrated to have mobility (Franken et al., 2001). The *drsG* gene itself it is not surrounded by any insertion sequences or mobile elements, though, and the mechanisms promoting the observed excision or insertion events are unclear. Its role as virulence factors in in certain SDSE associated *emm*-types remains to be elucidated.

In contrast to the clonally restricted virulence profiles of chromosomally encoded genes, virulence factors transferred by mobile elements appeared to have a random distribution (Figure 4B). Nevertheless, transduction of phages and conjugation of ICEs between bacterial cells play an important role in bacterial evolution and adaption, both for escaping selective pressure and as an additional means of horizontal genetic exchange and virulence enhancement. Screening of genomes for such mobile elements in our study (Figure 4B and Table 1) revealed a higher presence of ICEs with an average of 2.9 per SDSE genome, in contrast to a low prevalence of bacteriophages among the SDSE isolates (here 0.6 bacteriophages/genome). This was reflected in the high prevalence of the ICE-associated virulence factors *bspA* and the *alp*-gene family among our strains, and fewer phage-associated virulence factors (Table 1). In line with our findings, a comprehensive search for ICEs in streptococcal species by Ambroset et al. (2015) showed also a relatively higher prevalence of ICEs among *S. dysgalactiae* isolates (2.0 per genome), while the prevalence of ICEs in *S. pyogenes* remained low with an average of only 0.3 ICEs per genome. According to previous studies, the presence of particular cargo genes in ICEs protects the cells against other mobile elements. More specifically, in *Bacillus subtilis* for instance, the presence of a single gene in the integrative and conjugative element ICEBs1 was shown to have an important role in repressing the adaption of bacteriophages, providing selective pressure for the host cells to maintain the presence of ICEs (Johnson et al., 2022). An attractive hypothesis is therefore that the strong presence of ICEs in SDSE genomes might reduce the number of bacteriophages in the cells. Nevertheless, more studies on the interplay between phage, ICE and microbe are needed to explore the evolutionary contribution of mobile genetic elements in SDSE.

The confined geographic and temporal setting of our study limits the ability to study temporal dynamics of the SDSE population. Moreover, the study period of the WGS studies from Japan and Canada did not completely overlap that in our study, and a potential impact of temporal shifts in circulating genotypes cannot be ruled out. However, our study included all blood culture isolates in Norway in 2018, and as such provides a very accurate and comprehensive snapshot of the SDSE epidemiology in our country. The geographical differences in the distribution of clades across Norway might be random since a limited sample size and insufficient power, did not allow for any statistical assessment. Yet, the extensive similarities with *emm*- and ST-profiles previously published indicated that our results also reflect the epidemiologic situation in other European countries.

## Conclusion

The genetic landscape of SDSE appears to be continuously shaped by mutations and horizontal genetic transfer events, possibly driven by host-pathogen interactions and selection pressure. This genomic evolution fuels the temporal and geographic dynamics of the SDSE population, and gives rise to novel successful lineages such as the CC20_*stG62647* clade. Traditional genotypic markers often lack sufficient phylogenetic resolution to capture these evolutionary events, and WGS has become a powerful tool for elucidating bacterial population dynamics.

## Data availability statement

The datasets presented in this study can be found in online repositories. The names of the repository/repositories and accession number(s) can be found in the article/Supplementary material.

## Ethics statement

The studies involving human participants were reviewed and approved by Western Norway Regional Ethics Committee (2021/63132). Written informed consent for participation was not required for this study in accordance with the national legislation and the institutional requirements.

## Author contributions

MS, OO, and CMJ: conceptualization and supervision. AK and OO: methodology, software, and formal analysis. OO: data curation and visualization. AK: writing—original draft preparation. AK, CMJ, SS, AS, and OO: writing—review and editing. MS, SS, CMJ, and OO: project administration. All authors have read and agreed to the published version of the manuscript.

## The NORWEGIAN Study Group on *Streptococcus dysgalactiae* Members

Aasmund Fostervold, Department of Clinical Microbiology, Stavanger University Hospital, Stavanger, Norway; Aleksandra Jakovljev, Department of Microbiology, St. Olav’s University Hospital, Trondheim, Norway; Annette Onken, Department of Clinical Microbiology, Vestre Viken Hospital Trust, Baerum, Norway; Åshild Marvik, Department of Microbiology, Vestfold Hospital Trust, Tønsberg, Norway; Einar Nilsen, Department of Microbiology, Møre and Romsdal Hospital Trust, Ålesund, Norway; Fredrik Müller, Department of Microbiology, Oslo University Hospital, Oslo, Norway; Ghantous Milad Chedid, Laboratory for Clinical Microbiology, Fonna Hospital Trust, Haugesund, Norway; Gunnar Skov Simonsen, Department of Clinical Microbiology, University Hospital of North Norway, Tromsø, Norway; Kyriakos Zaragkoulias, Department of Clinical Microbiology, Nord-Trøndelag Hospital Trust, Levanger, Norway; Reidar Hjetland, Department of Microbiology, Division of Medicine, District General Hospital of Førde, Førde, Norway; Roar Magne Baevre-Jensen, Department of Clinical Microbiology, Vestre Viken Hospital Trust, Drammen, Norway; Sandra Åsheim, Unit for Clinical Microbiology, Norland Hospital Trust, Bodø, Norway; Ståle Tofteland, Department of Clinical Microbiology, Sørlandet Hospital Trust, Agder, Norway; Tine Smedsund Dons, Department of Clinical Microbiology, Innlandet Hospital Trust, Lillehammer, Norway; Truls Michael Leegaard, Department of Microbiology and Infection Control, Akerhus University Hospital, Lørenskog, Norway.

## Footnotes

1
https://www.ssb.no/

2
https://www.cdc.gov/streplab/groupa-strep/emm-background.html

3
https://pubmlst.org/

4
https://cge.cbs.dtu.dk/services

5
http://www.ebi.ac.uk/ena/data/view/ERS1066625

6
www.mgc.ac.cn/VFs

## Supplementary material

Supplementary Figure 1 Whole genome single-nucleotide polymorphism (SNP)-derived phylogenetic tree of invasive *Streptococcus dysgalactiae* subspecies *equisimilis* (SDSE) obtained in Norway during 2018. *N* = 274. Here the canine-associated genome *stC9431* is included and highlighted in red. This tree was constructed with CSI phylogeny website. Scale bar indicates the estimated evolutionary divergence between isolates measured in substitution per site.

Supplementary Figure 2 The Fibronectin, collagen and T-antigen (FCT) region, also known as Pilus island-1 region, harboring the *gfba* adhesin observed between two phylogenetically closely related isolates. The comparison was performed using Geneious alignment. The FCT-region in each *Streptococcus dysgalactiae* subspecies *equisimilis* (SDSE) isolate is highlighted in orange. The gray shaded areas indicate 100% homology, and blue lines in the alignment indicate single nucleotide polymorphisms. The scale indicates number of base pairs.
