# Supplementary figures and images for: Genomic epidemiology of Streptococcus dysgalactiae subsp. equisimilis strains causing invasive disease in Norway during 2018
Source: Front Microbiol. 2023 Apr 17;14:1171913. doi: 10.3389/fmicb.2023.1171913 (PMC10361778; doi:10.3389/fmicb.2023.1171913)

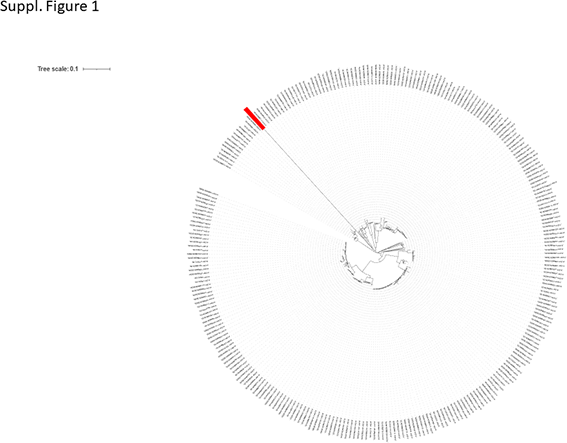

Supplement: Supplementary Figure 1 — Whole genome single-nucleotide polymorphism (SNP)-derived phylogenetic tree of invasive Streptococcus dysgalactiae subspecies equisimilis (SDSE) obtained in Norway during 2018. N = 274. Here the canine-associated genome stC9431 is included and highlighted in red. This tree was constructed with CSI phylogeny website. Scale bar indicates the estimated evolutionary divergence between isolates measured in substitution per site. [file Image_1.tif]

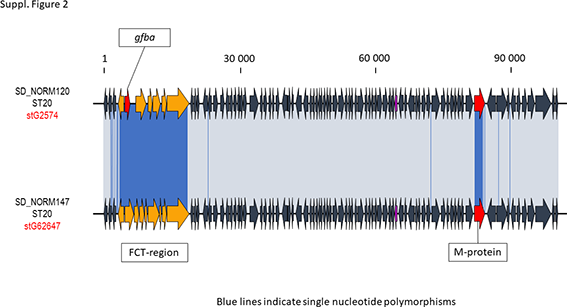

Supplement: Supplementary Figure 2 — The Fibronectin, collagen and T-antigen (FCT) region, also known as Pilus island-1 region, harboring the gfba adhesin observed between two phylogenetically closely related isolates. The comparison was performed using Geneious alignment. The FCT-region in each Streptococcus dysgalactiae subspecies equisimilis (SDSE) isolate is highlighted in orange. The gray shaded areas indicate 100% homology, and blue lines in the alignment indicate single nucleotide polymorphisms. The scale indicates number of base pairs. [file Image_2.tif]
